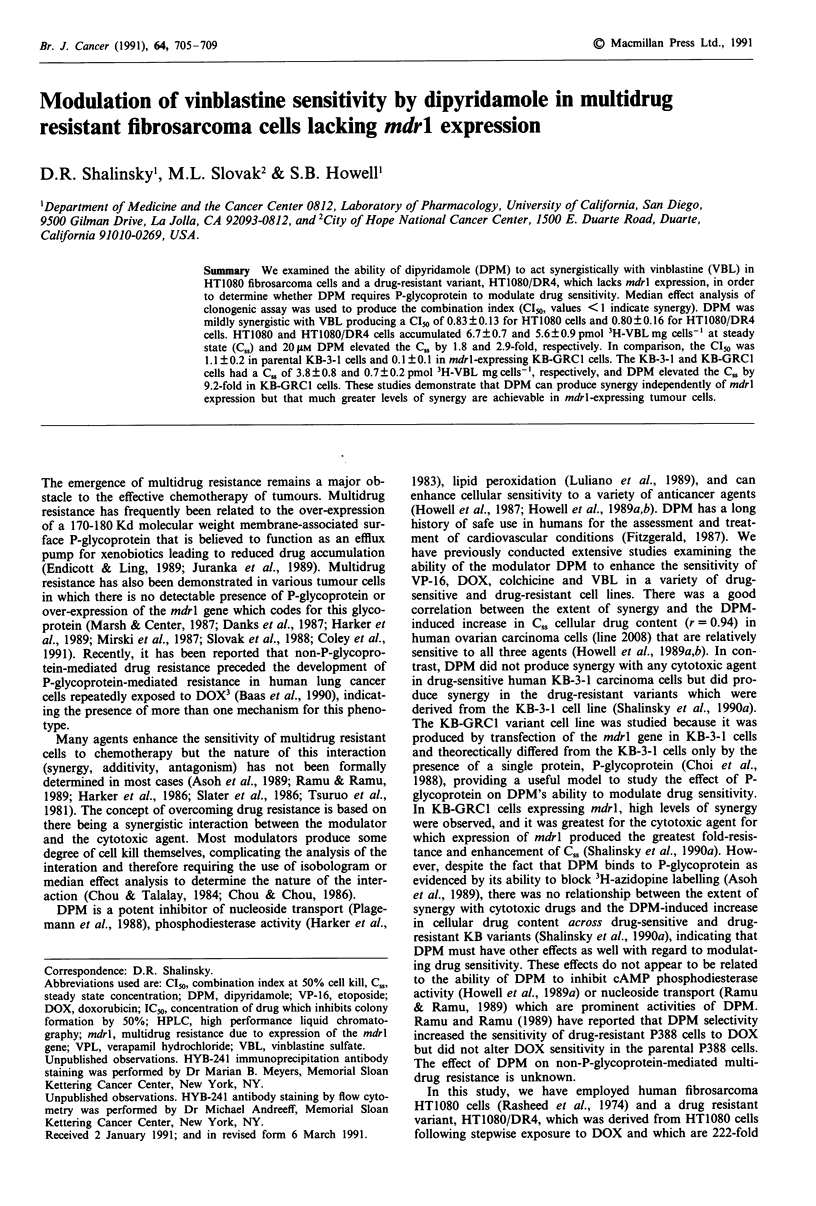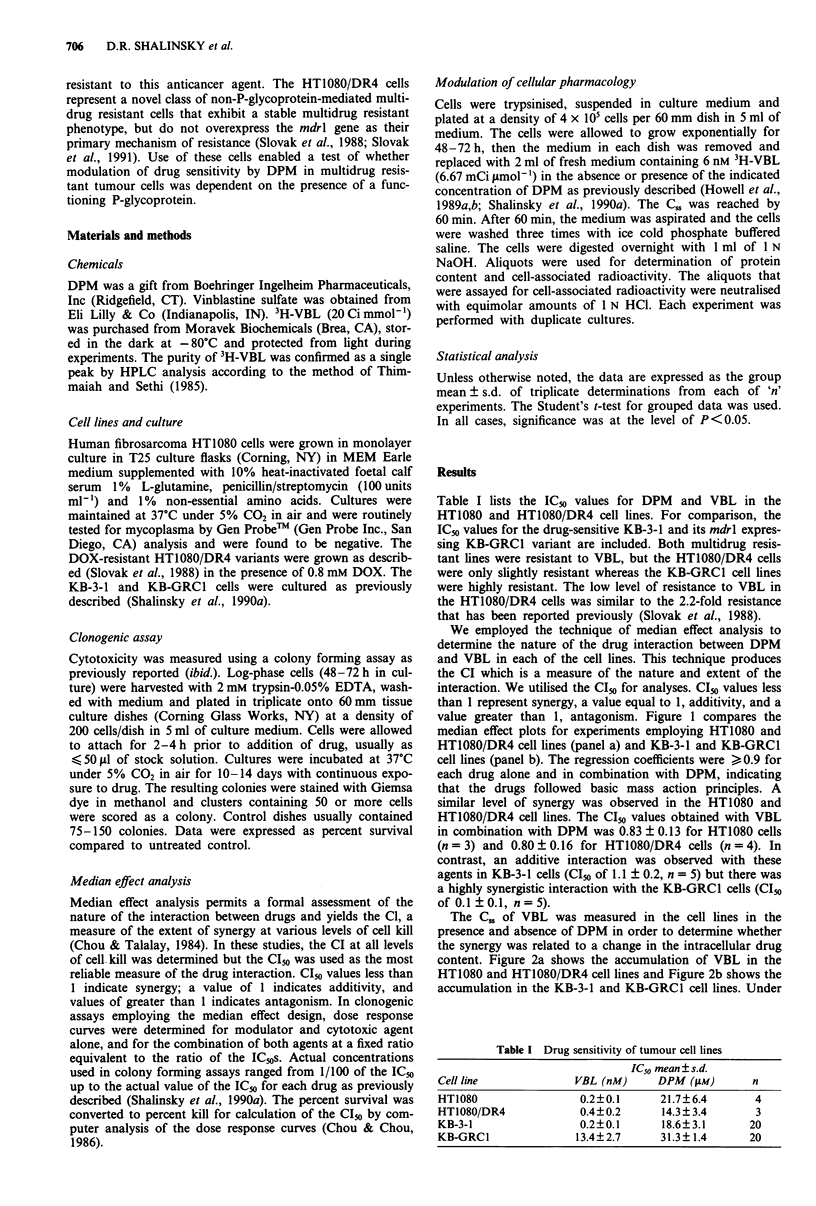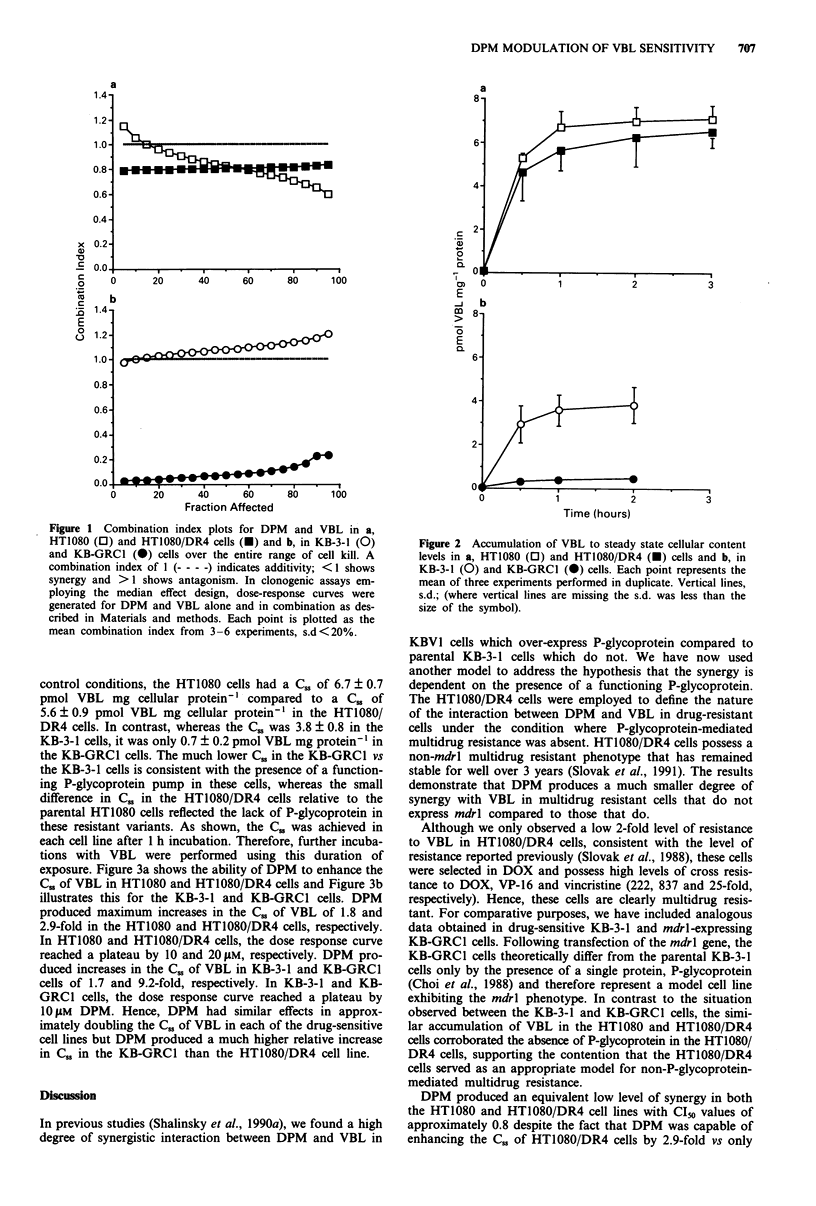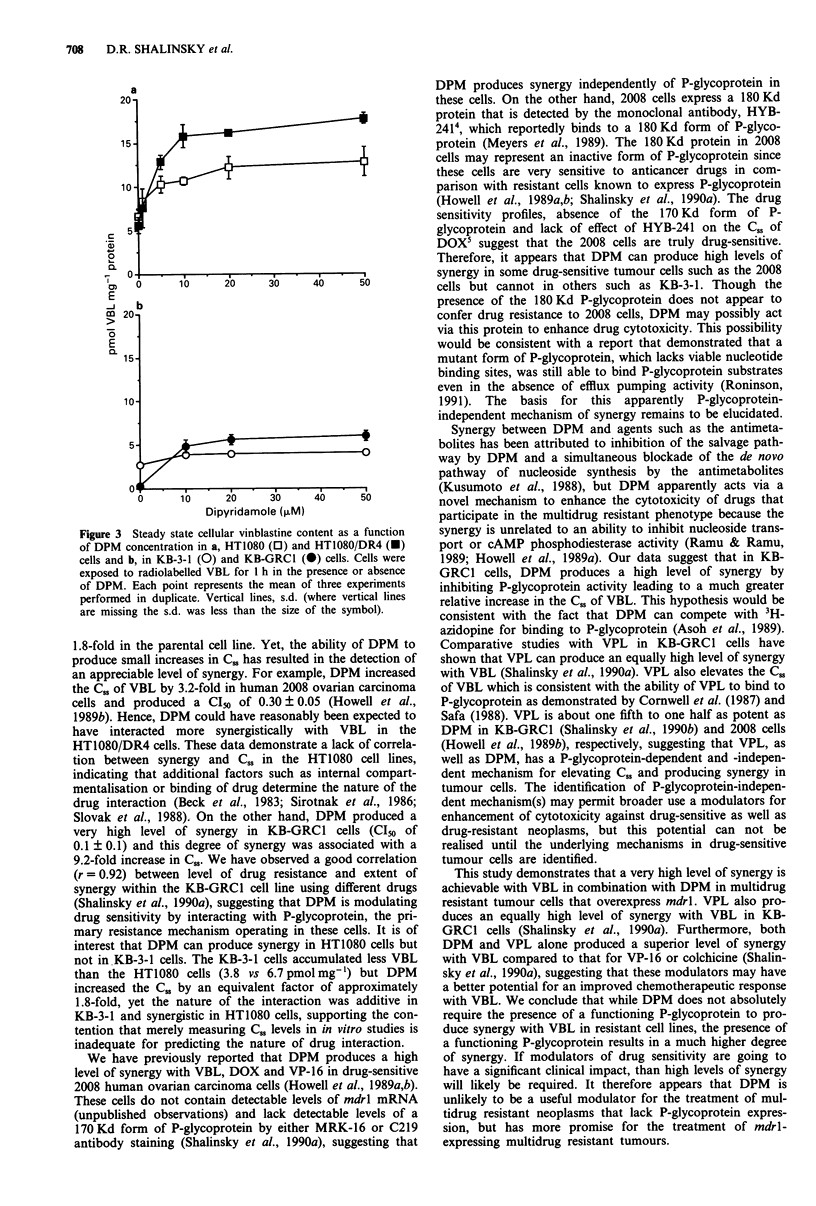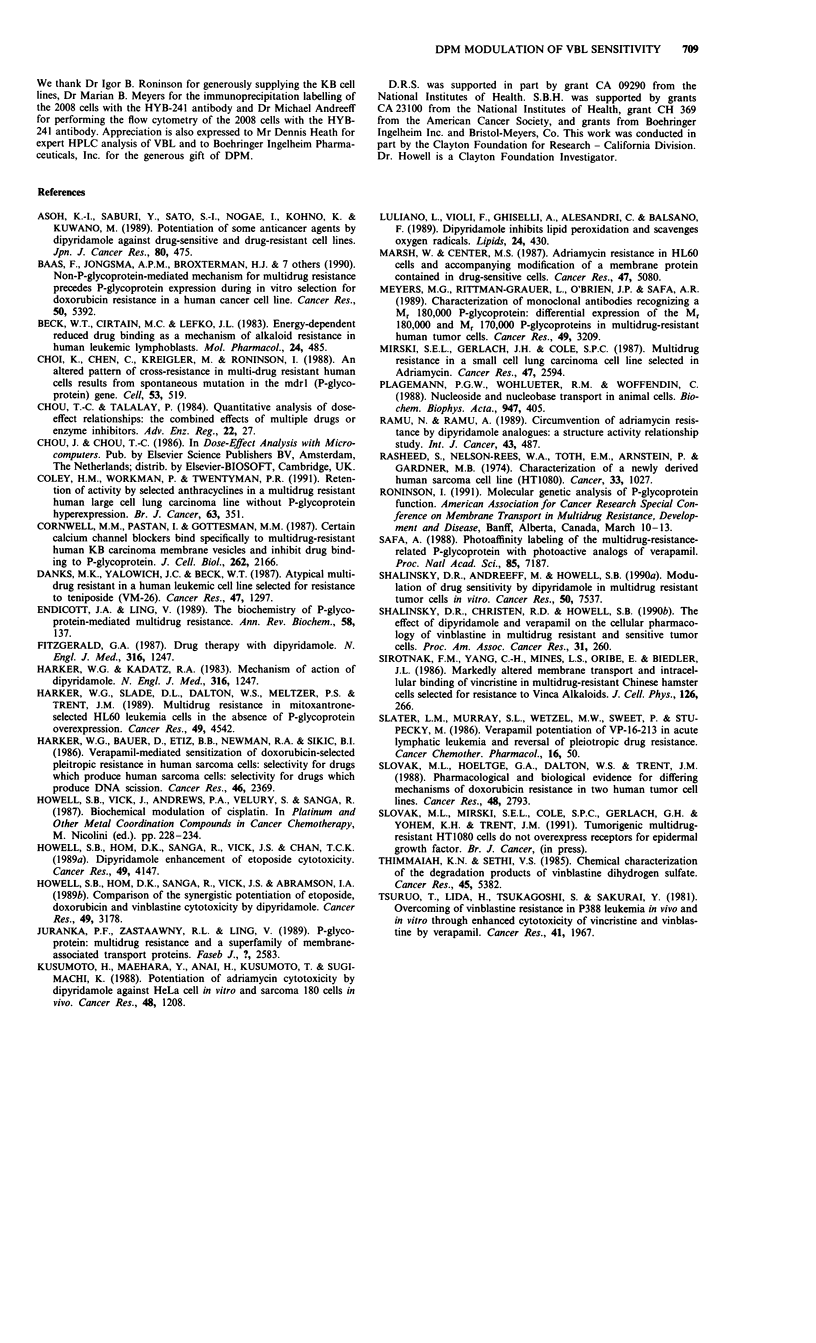# Modulation of vinblastine sensitivity by dipyridamole in multidrug resistant fibrosarcoma cells lacking mdr1 expression.

**DOI:** 10.1038/bjc.1991.385

**Published:** 1991-10

**Authors:** D. R. Shalinsky, M. L. Slovak, S. B. Howell

**Affiliations:** Department of Medicine, University of California, San Diego, La Jolla 92093-0812.

## Abstract

We examined the ability of dipyridamole (DPM) to act synergistically with vinblastine (VBL) in HT1080 fibrosarcoma cells and a drug-resistant variant, HT1080/DR4, which lacks mdr1 expression, in order to determine whether DPM requires P-glycoprotein to modulate drug sensitivity. Median effect analysis of clonogenic assay was used to produce the combination index (CI50, values less than 1 indicate synergy). DPM was mildly synergistic with VBL producing a CI50 of 0.83 +/- 0.13 for HT1080 cells and 0.80 +/- 0.16 for HT1080/DR4 cells. HT1080 and HT1080/DR4 cells accumulated 6.7 +/- 0.7 and 5.6 +/- 0.9 pmol 3H-VBL mg cells-1 at steady state (Css) and 20 microM DPM elevated the Css by 1.8 and 2.9-fold, respectively. In comparison, the CI50 was 1.1 +/- 0.2 in parental KB-3-1 cells and 0.1 +/- 0.1 in mdr1-expressing KB-GRC1 cells. The KB-3-1 and KB-GRC1 cells had a Css of 3.8 +/- 0.8 and 0.7 +/- 0.2 pmol 3H-VBL mg cells-1, respectively, and DPM elevated the Css by 9.2-fold in KB-GRC1 cells. These studies demonstrate that DPM can produce synergy independently of mdr1 expression but that much greater levels of synergy are achievable in mdr1-expressing tumour cells.


Br. J. Cancer (1991), 64, 705-709 © Macmillan Press Ltd., 1991

### Modulation of vinblastine sensitivity by dipyridamole in multidrug resistant fibrosarcoma cells lacking *mdr*1 expression

D.R. Shalinsky[1], M.L. Slovak[2] & S.B. Howell[1]

[1]*Department of Medicine and the Cancer Center 0812, Laboratory of Pharmacology, University of California, San Diego, 9500 Gilman Drive, La Jolla, CA 92093-0812, and* [2]*City of Hope National Cancer Center, 1500 E. Duarte Road, Duarte, California 91010-0269, USA.*

**Summary** We examined the ability of dipyridamole (DPM) to act synergistically with vinblastine (VBL) in HT1080 fibrosarcoma cells and a drug-resistant variant, HT1080/DR4, which lacks *mdr*1 expression, in order to determine whether DPM requires P-glycoprotein to modulate drug sensitivity. Median effect analysis of clonogenic assay was used to produce the combination index ($CI_{50}$, values $<1$ indicate synergy). DPM was mildly synergistic with VBL producing a $CI_{50}$ of $0.83 \pm 0.13$ for HT1080 cells and $0.80 \pm 0.16$ for HT1080/DR4 cells. HT1080 and HT1080/DR4 cells accumulated $6.7 \pm 0.7$ and $5.6 \pm 0.9$ pmol $^3$H-VBL mg cells$^{-1}$ at steady state ($C_{ss}$) and 20 $\mu$M DPM elevated the $C_{ss}$ by 1.8 and 2.9-fold, respectively. In comparison, the $CI_{50}$ was $1.1 \pm 0.2$ in parental KB-3-1 cells and $0.1 \pm 0.1$ in *mdr*1-expressing KB-GRC1 cells. The KB-3-1 and KB-GRC1 cells had a $C_{ss}$ of $3.8 \pm 0.8$ and $0.7 \pm 0.2$ pmol $^3$H-VBL mg cells$^{-1}$, respectively, and DPM elevated the $C_{ss}$ by 9.2-fold in KB-GRC1 cells. These studies demonstrate that DPM can produce synergy independently of *mdr*1 expression but that much greater levels of synergy are achievable in *mdr*1-expressing tumour cells.

The emergence of multidrug resistance remains a major obstacle to the effective chemotherapy of tumours. Multidrug resistance has frequently been related to the over-expression of a 170-180 Kd molecular weight membrane-associated surface P-glycoprotein that is believed to function as an efflux pump for xenobiotics leading to reduced drug accumulation (Endicott & Ling, 1989; Juranka *et al.*, 1989). Multidrug resistance has also been demonstrated in various tumour cells in which there is no detectable presence of P-glycoprotein or over-expression of the *mdr*1 gene which codes for this glycoprotein (Marsh & Center, 1987; Danks *et al.*, 1987; Harker *et al.*, 1989; Mirski *et al.*, 1987; Slovak *et al.*, 1988; Coley *et al.*, 1991). Recently, it has been reported that non-P-glycoprotein-mediated drug resistance preceded the development of P-glycoprotein-mediated resistance in human lung cancer cells repeatedly exposed to DOX[3] (Baas *et al.*, 1990), indicating the presence of more than one mechanism for this phenotype.

Many agents enhance the sensitivity of multidrug resistant cells to chemotherapy but the nature of this interaction (synergy, additivity, antagonism) has not been formally determined in most cases (Asoh *et al.*, 1989; Ramu & Ramu, 1989; Harker *et al.*, 1986; Slater *et al.*, 1986; Tsuruo *et al.*, 1981). The concept of overcoming drug resistance is based on there being a synergistic interaction between the modulator and the cytotoxic agent. Most modulators produce some degree of cell kill themselves, complicating the analysis of the interation and therefore requiring the use of isobologram or median effect analysis to determine the nature of the interaction (Chou & Talalay, 1984; Chou & Chou, 1986).

DPM is a potent inhibitor of nucleoside transport (Plagemann *et al.*, 1988), phosphodiesterase activity (Harker *et al.*, 1983), lipid peroxidation (Luliano *et al.*, 1989), and can enhance cellular sensitivity to a variety of anticancer agents (Howell *et al.*, 1987; Howell *et al.*, 1989*a*,*b*). DPM has a long history of safe use in humans for the assessment and treatment of cardiovascular conditions (Fitzgerald, 1987). We have previously conducted extensive studies examining the ability of the modulator DPM to enhance the sensitivity of VP-16, DOX, colchicine and VBL in a variety of drug-sensitive and drug-resistant cell lines. There was a good correlation between the extent of synergy and the DPM-induced increase in $C_{ss}$ cellular drug content ($r = 0.94$) in human ovarian carcinoma cells (line 2008) that are relatively sensitive to all three agents (Howell *et al.*, 1989*a*,*b*). In contrast, DPM did not produce synergy with any cytotoxic agent in drug-sensitive human KB-3-1 carcinoma cells but did produce synergy in the drug-resistant variants which were derived from the KB-3-1 cell line (Shalinsky *et al.*, 1990*a*). The KB-GRC1 variant cell line was studied because it was produced by transfection of the *mdr*1 gene in KB-3-1 cells and theorectically differed from the KB-3-1 cells only by the presence of a single protein, P-glycoprotein (Choi *et al.*, 1988), providing a useful model to study the effect of P-glycoprotein on DPM's ability to modulate drug sensitivity. In KB-GRC1 cells expressing *mdr*1, high levels of synergy were observed, and it was greatest for the cytotoxic agent for which expression of *mdr*1 produced the greatest fold-resistance and enhancement of $C_{ss}$ (Shalinsky *et al.*, 1990*a*). However, despite the fact that DPM binds to P-glycoprotein as evidenced by its ability to block $^3$H-azidopine labelling (Asoh *et al.*, 1989), there was no relationship between the extent of synergy with cytotoxic drugs and the DPM-induced increase in cellular drug content *across* drug-sensitive and drug-resistant KB variants (Shalinsky *et al.*, 1990*a*), indicating that DPM must have other effects as well with regard to modulating drug sensitivity. These effects do not appear to be related to the ability of DPM to inhibit cAMP phosphodiesterase activity (Howell *et al.*, 1989*a*) or nucleoside transport (Ramu & Ramu, 1989) which are prominent activities of DPM. Ramu and Ramu (1989) have reported that DPM selectivity increased the sensitivity of drug-resistant P388 cells to DOX but did not alter DOX sensitivity in the parental P388 cells. The effect of DPM on non-P-glycoprotein-mediated multidrug resistance is unknown.

In this study, we have employed human fibrosarcoma HT1080 cells (Rasheed *et al.*, 1974) and a drug resistant variant, HT1080/DR4, which was derived from HT1080 cells following stepwise exposure to DOX and which are 222-fold

Correspondence: D.R. Shalinsky.

Abbreviations used are: $CI_{50}$, combination index at 50% cell kill, $C_{ss}$, steady state concentration; DPM, dipyridamole; VP-16, etoposide; DOX, doxorubicin; $IC_{50}$, concentration of drug which inhibits colony formation by 50%; HPLC, high performance liquid chromatography; *mdr*1, multidrug resistance due to expression of the *mdr*1 gene; VPL, verapamil hydrochloride; VBL, vinblastine sulfate.

Unpublished observations. HYB-241 immunoprecipitation antibody staining was performed by Dr Marian B. Meyers, Memorial Sloan Kettering Cancer Center, New York, NY.

Unpublished observations. HYB-241 antibody staining by flow cytometry was performed by Dr Michael Andreeff, Memorial Sloan Kettering Cancer Center, New York, NY.

Received 2 January 1991; and in revised form 6 March 1991.

resistant to this anticancer agent. The HT1080/DR4 cells represent a novel class of non-P-glycoprotein-mediated multidrug resistant cells that exhibit a stable multidrug resistant phenotype, but do not overexpress the *mdr*1 gene as their primary mechanism of resistance (Slovak *et al.*, 1988; Slovak *et al.*, 1991). Use of these cells enabled a test of whether modulation of drug sensitivity by DPM in multidrug resistant tumour cells was dependent on the presence of a functioning P-glycoprotein.

### Materials and methods

### Chemicals

DPM was a gift from Boehringer Ingelheim Pharmaceuticals, Inc (Ridgefield, CT). Vinblastine sulfate was obtained from Eli Lilly & Co (Indianapolis, IN). $^3$H-VBL (20 Ci mmol$^{-1}$) was purchased from Moravek Biochemicals (Brea, CA), stored in the dark at −80°C and protected from light during experiments. The purity of $^3$H-VBL was confirmed as a single peak by HPLC analysis according to the method of Thimmaiah and Sethi (1985).

### Cell lines and culture

Human fibrosarcoma HT1080 cells were grown in monolayer culture in T25 culture flasks (Corning, NY) in MEM Earle medium supplemented with 10% heat-inactivated foetal calf serum 1% L-glutamine, penicillin/streptomycin (100 units ml$^{-1}$) and 1% non-essential amino acids. Cultures were maintained at 37°C under 5% $CO_2$ in air and were routinely tested for mycoplasma by Gen Probe™ (Gen Probe Inc., San Diego, CA) analysis and were found to be negative. The DOX-resistant HT1080/DR4 variants were grown as described (Slovak *et al.*, 1988) in the presence of 0.8 mM DOX. The KB-3-1 and KB-GRC1 cells were cultured as previously described (Shalinsky *et al.*, 1990*a*).

### Clonogenic assay

Cytotoxicity was measured using a colony forming assay as previously reported (*ibid.*). Log-phase cells (48–72 h in culture) were harvested with 2 mM trypsin-0.05% EDTA, washed with medium and plated in triplicate onto 60 mm tissue culture dishes (Corning Glass Works, NY) at a density of 200 cells/dish in 5 ml of culture medium. Cells were allowed to attach for 2–4 h prior to addition of drug, usually as $\leqslant 50\,\mu$l of stock solution. Cultures were incubated at 37°C under 5% $CO_2$ in air for 10–14 days with continuous exposure to drug. The resulting colonies were stained with Giemsa dye in methanol and clusters containing 50 or more cells were scored as a colony. Control dishes usually contained 75–150 colonies. Data were expressed as percent survival compared to untreated control.

### Median effect analysis

Median effect analysis permits a formal assessment of the nature of the interaction between drugs and yields the Cl, a measure of the extent of synergy at various levels of cell kill (Chou & Talalay, 1984). In these studies, the CI at all levels of cell kill was determined but the $CI_{50}$ was used as the most reliable measure of the drug interaction. $CI_{50}$ values less than 1 indicate synergy; a value of 1 indicates additivity, and values of greater than 1 indicates antagonism. In clonogenic assays employing the median effect design, dose response curves were determined for modulator and cytotoxic agent alone, and for the combination of both agents at a fixed ratio equivalent to the ratio of the $IC_{50}$s. Actual concentrations used in colony forming assays ranged from 1/100 of the $IC_{50}$ up to the actual value of the $IC_{50}$ for each drug as previously described (Shalinsky *et al.*, 1990*a*). The percent survival was converted to percent kill for calculation of the $CI_{50}$ by computer analysis of the dose response curves (Chou & Chou, 1986).

### Modulation of cellular pharmacology

Cells were trypsinised, suspended in culture medium and plated at a density of $4 \times 10^5$ cells per 60 mm dish in 5 ml of medium. The cells were allowed to grow exponentially for 48–72 h, then the medium in each dish was removed and replaced with 2 ml of fresh medium containing 6 nM $^3$H-VBL (6.67 mCi $\mu$mol$^{-1}$) in the absence or presence of the indicated concentration of DPM as previously described (Howell *et al.*, 1989*a*,*b*; Shalinsky *et al.*, 1990*a*). The $C_{ss}$ was reached by 60 min. After 60 min, the medium was aspirated and the cells were washed three times with ice cold phosphate buffered saline. The cells were digested overnight with 1 ml of 1 N NaOH. Aliquots were used for determination of protein content and cell-associated radioactivity. The aliquots that were assayed for cell-associated radioactivity were neutralised with equimolar amounts of 1 N HCl. Each experiment was performed with duplicate cultures.

### Statistical analysis

Unless otherwise noted, the data are expressed as the group mean ± s.d. of triplicate determinations from each of '*n*' experiments. The Student's *t*-test for grouped data was used. In all cases, significance was at the level of $P < 0.05$.

### Results

Table I lists the $IC_{50}$ values for DPM and VBL in the HT1080 and HT1080/DR4 cell lines. For comparison, the $IC_{50}$ values for the drug-sensitive KB-3-1 and its *mdr*1 expressing KB-GRC1 variant are included. Both multidrug resistant lines were resistant to VBL, but the HT1080/DR4 cells were only slightly resistant whereas the KB-GRC1 cell lines were highly resistant. The low level of resistance to VBL in the HT1080/DR4 cells was similar to the 2.2-fold resistance that has been reported previously (Slovak *et al.*, 1988).

We employed the technique of median effect analysis to determine the nature of the drug interaction between DPM and VBL in each of the cell lines. This technique produces the CI which is a measure of the nature and extent of the interaction. We utilised the $CI_{50}$ for analyses. $CI_{50}$ values less than 1 represent synergy, a value equal to 1, additivity, and a value greater than 1, antagonism. Figure 1 compares the median effect plots for experiments employing HT1080 and HT1080/DR4 cell lines (panel a) and KB-3-1 and KB-GRC1 cell lines (panel b). The regression coefficients were $\geqslant 0.9$ for each drug alone and in combination with DPM, indicating that the drugs followed basic mass action principles. A similar level of synergy was observed in the HT1080 and HT1080/DR4 cell lines. The $CI_{50}$ values obtained with VBL in combination with DPM was $0.83 \pm 0.13$ for HT1080 cells ($n = 3$) and $0.80 \pm 0.16$ for HT1080/DR4 cells ($n = 4$). In contrast, an additive interaction was observed with these agents in KB-3-1 cells ($CI_{50}$ of $1.1 \pm 0.2$, $n = 5$) but there was a highly synergistic interaction with the KB-GRC1 cells ($CI_{50}$ of $0.1 \pm 0.1$, $n = 5$).

The $C_{ss}$ of VBL was measured in the cell lines in the presence and absence of DPM in order to determine whether the synergy was related to a change in the intracellular drug content. Figure 2a shows the accumulation of VBL in the HT1080 and HT1080/DR4 cell lines and Figure 2b shows the accumulation in the KB-3-1 and KB-GRC1 cell lines. Under

**Table I** Drug sensitivity of tumour cell lines

| Cell line | $IC_{50}$ mean ± s.d. VBL (nM) | DPM (μM) | n |
|---|---|---|---|
| HT1080 | 0.2±0.1 | 21.7±6.4 | 4 |
| HT1080/DR4 | 0.4±0.2 | 14.3±3.4 | 3 |
| KB-3-1 | 0.2±0.1 | 18.6±3.1 | 20 |
| KB-GRC1 | 13.4±2.7 | 31.3±1.4 | 20 |

**Figure 1** Combination index plots for DPM and VBL in **a**, HT1080 (□) and HT1080/DR4 cells (■) and **b**, in KB-3-1 (○) and KB-GRC1 (●) cells over the entire range of cell kill. A combination index of 1 (- - - -) indicates additivity; <1 shows synergy and >1 shows antagonism. In clonogenic assays employing the median effect design, dose-response curves were generated for DPM and VBL alone and in combination as described in Materials and methods. Each point is plotted as the mean combination index from 3–6 experiments, s.d<20%.

**Figure 2** Accumulation of VBL to steady state cellular content levels in **a**, HT1080 (□) and HT1080/DR4 (■) cells and **b**, in KB-3-1 (○) and KB-GRC1 (●) cells. Each point represents the mean of three experiments performed in duplicate. Vertical lines, s.d.; (where vertical lines are missing the s.d. was less than the size of the symbol).

control conditions, the HT1080 cells had a $C_{ss}$ of 6.7 ± 0.7 pmol VBL mg cellular protein$^{-1}$ compared to a $C_{ss}$ of 5.6 ± 0.9 pmol VBL mg cellular protein$^{-1}$ in the HT1080/DR4 cells. In contrast, whereas the $C_{ss}$ was 3.8 ± 0.8 in the KB-3-1 cells, it was only 0.7 ± 0.2 pmol VBL mg protein$^{-1}$ in the KB-GRC1 cells. The much lower $C_{ss}$ in the KB-GRC1 *vs* the KB-3-1 cells is consistent with the presence of a functioning P-glycoprotein pump in these cells, whereas the small difference in $C_{ss}$ in the HT1080/DR4 cells relative to the parental HT1080 cells reflected the lack of P-glycoprotein in these resistant variants. As shown, the $C_{ss}$ was achieved in each cell line after 1 h incubation. Therefore, further incubations with VBL were performed using this duration of exposure. Figure 3a shows the ability of DPM to enhance the $C_{ss}$ of VBL in HT1080 and HT1080/DR4 cells and Figure 3b illustrates this for the KB-3-1 and KB-GRC1 cells. DPM produced maximum increases in the $C_{ss}$ of VBL of 1.8 and 2.9-fold in the HT1080 and HT1080/DR4 cells, respectively. In HT1080 and HT1080/DR4 cells, the dose response curve reached a plateau by 10 and 20 μM, respectively. DPM produced increases in the $C_{ss}$ of VBL in KB-3-1 and KB-GRC1 cells of 1.7 and 9.2-fold, respectively. In KB-3-1 and KB-GRC1 cells, the dose response curve reached a plateau by 10 μM DPM. Hence, DPM had similar effects in approximately doubling the $C_{ss}$ of VBL in each of the drug-sensitive cell lines but DPM produced a much higher relative increase in $C_{ss}$ in the KB-GRC1 than the HT1080/DR4 cell line.

### Discussion

In previous studies (Shalinsky *et al.*, 1990*a*), we found a high degree of synergistic interaction between DPM and VBL in KBV1 cells which over-express P-glycoprotein compared to parental KB-3-1 cells which do not. We have now used another model to address the hypothesis that the synergy is dependent on the presence of a functioning P-glycoprotein. The HT1080/DR4 cells were employed to define the nature of the interaction between DPM and VBL in drug-resistant cells under the condition where P-glycoprotein-mediated multidrug resistance was absent. HT1080/DR4 cells possess a non-*mdr*1 multidrug resistant phenotype that has remained stable for well over 3 years (Slovak *et al.*, 1991). The results demonstrate that DPM produces a much smaller degree of synergy with VBL in multidrug resistant cells that do not express *mdr*1 compared to those that do.

Although we only observed a low 2-fold level of resistance to VBL in HT1080/DR4 cells, consistent with the level of resistance reported previously (Slovak *et al.*, 1988), these cells were selected in DOX and possess high levels of cross resistance to DOX, VP-16 and vincristine (222, 837 and 25-fold, respectively). Hence, these cells are clearly multidrug resistant. For comparative purposes, we have included analogous data obtained in drug-sensitive KB-3-1 and *mdr*1-expressing KB-GRC1 cells. Following transfection of the *mdr*1 gene, the KB-GRC1 cells theoretically differ from the parental KB-3-1 cells only by the presence of a single protein, P-glycoprotein (Choi *et al.*, 1988) and therefore represent a model cell line exhibiting the *mdr*1 phenotype. In contrast to the situation observed between the KB-3-1 and KB-GRC1 cells, the similar accumulation of VBL in the HT1080 and HT1080/DR4 cells corroborated the absence of P-glycoprotein in the HT1080/DR4 cells, supporting the contention that the HT1080/DR4 cells served as an appropriate model for non-P-glycoprotein-mediated multidrug resistance.

DPM produced an equivalent low level of synergy in both the HT1080 and HT1080/DR4 cell lines with $CI_{50}$ values of approximately 0.8 despite the fact that DPM was capable of enhancing the $C_{ss}$ of HT1080/DR4 cells by 2.9-fold *vs* only

**Figure 3** Steady state cellular vinblastine content as a function of DPM concentration in **a**, HT1080 (□) and HT1080/DR4 (■) cells and **b**, in KB-3-1 (○) and KB-GRC1 (●) cells. Cells were exposed to radiolabelled VBL for 1 h in the presence or absence of DPM. Each point represents the mean of three experiments performed in duplicate. Vertical lines, s.d. (where vertical lines are missing the s.d. was less than the size of the symbol).

1.8-fold in the parental cell line. Yet, the ability of DPM to produce small increases in $C_{ss}$ has resulted in the detection of an appreciable level of synergy. For example, DPM increased the $C_{ss}$ of VBL by 3.2-fold in human 2008 ovarian carcinoma cells and produced a $CI_{50}$ of 0.30 ± 0.05 (Howell *et al.*, 1989*b*). Hence, DPM could have reasonably been expected to have interacted more synergistically with VBL in the HT1080/DR4 cells. These data demonstrate a lack of correlation between synergy and $C_{ss}$ in the HT1080 cell lines, indicating that additional factors such as internal compartmentalisation or binding of drug determine the nature of the drug interaction (Beck *et al.*, 1983; Sirotnak *et al.*, 1986; Slovak *et al.*, 1988). On the other hand, DPM produced a very high level of synergy in KB-GRC1 cells ($CI_{50}$ of 0.1 ± 0.1) and this degree of synergy was associated with a 9.2-fold increase in $C_{ss}$. We have observed a good correlation ($r = 0.92$) between level of drug resistance and extent of synergy within the KB-GRC1 cell line using different drugs (Shalinsky *et al.*, 1990*a*), suggesting that DPM is modulating drug sensitivity by interacting with P-glycoprotein, the primary resistance mechanism operating in these cells. It is of interest that DPM can produce synergy in HT1080 cells but not in KB-3-1 cells. The KB-3-1 cells accumulated less VBL than the HT1080 cells (3.8 *vs* 6.7 pmol $mg^{-1}$) but DPM increased the $C_{ss}$ by an equivalent factor of approximately 1.8-fold, yet the nature of the interaction was additive in KB-3-1 and synergistic in HT1080 cells, supporting the contention that merely measuring $C_{ss}$ levels in *in vitro* studies is inadequate for predicting the nature of drug interaction.

We have previously reported that DPM produces a high level of synergy with VBL, DOX and VP-16 in drug-sensitive 2008 human ovarian carcinoma cells (Howell *et al.*, 1989*a,b*). These cells do not contain detectable levels of *mdr*1 mRNA (unpublished observations) and lack detectable levels of a 170 Kd form of P-glycoprotein by either MRK-16 or C219 antibody staining (Shalinsky *et al.*, 1990*a*), suggesting that DPM produces synergy independently of P-glycoprotein in these cells. On the other hand, 2008 cells express a 180 Kd protein that is detected by the monoclonal antibody, HYB-241[4], which reportedly binds to a 180 Kd form of P-glycoprotein (Meyers *et al.*, 1989). The 180 Kd protein in 2008 cells may represent an inactive form of P-glycoprotein since these cells are very sensitive to anticancer drugs in comparison with resistant cells known to express P-glycoprotein (Howell *et al.*, 1989*a,b*; Shalinsky *et al.*, 1990*a*). The drug sensitivity profiles, absence of the 170 Kd form of P-glycoprotein and lack of effect of HYB-241 on the $C_{ss}$ of DOX[5] suggest that the 2008 cells are truly drug-sensitive. Therefore, it appears that DPM can produce high levels of synergy in some drug-sensitive tumour cells such as the 2008 cells but cannot in others such as KB-3-1. Though the presence of the 180 Kd P-glycoprotein does not appear to confer drug resistance to 2008 cells, DPM may possibly act via this protein to enhance drug cytotoxicity. This possibility would be consistent with a report that demonstrated that a mutant form of P-glycoprotein, which lacks viable nucleotide binding sites, was still able to bind P-glycoprotein substrates even in the absence of efflux pumping activity (Roninson, 1991). The basis for this apparently P-glycoprotein-independent mechanism of synergy remains to be elucidated.

Synergy between DPM and agents such as the antimetabolites has been attributed to inhibition of the salvage pathway by DPM and a simultaneous blockade of the *de novo* pathway of nucleoside synthesis by the antimetabolites (Kusumoto *et al.*, 1988), but DPM apparently acts via a novel mechanism to enhance the cytotoxicity of drugs that participate in the multidrug resistant phenotype because the synergy is unrelated to an ability to inhibit nucleoside transport or cAMP phosphodiesterase activity (Ramu & Ramu, 1989; Howell *et al.*, 1989*a*). Our data suggest that in KB-GRC1 cells, DPM produces a high level of synergy by inhibiting P-glycoprotein activity leading to a much greater relative increase in the $C_{ss}$ of VBL. This hypothesis would be consistent with the fact that DPM can compete with $^3$H-azidopine for binding to P-glycoprotein (Asoh *et al.*, 1989). Comparative studies with VPL in KB-GRC1 cells have shown that VPL can produce an equally high level of synergy with VBL (Shalinsky *et al.*, 1990*a*). VPL also elevates the $C_{ss}$ of VBL which is consistent with the ability of VPL to bind to P-glycoprotein as demonstrated by Cornwell *et al.* (1987) and Safa (1988). VPL is about one fifth to one half as potent as DPM in KB-GRC1 (Shalinsky *et al.*, 1990*b*) and 2008 cells (Howell *et al.*, 1989*b*), respectively, suggesting that VPL, as well as DPM, has a P-glycoprotein-dependent and -independent mechanism for elevating $C_{ss}$ and producing synergy in tumour cells. The identification of P-glycoprotein-independent mechanism(s) may permit broader use a modulators for enhancement of cytotoxicity against drug-sensitive as well as drug-resistant neoplasms, but this potential can not be realised until the underlying mechanisms in drug-sensitive tumour cells are identified.

This study demonstrates that a very high level of synergy is achievable with VBL in combination with DPM in multidrug resistant tumour cells that overexpress *mdr*1. VPL also produces an equally high level of synergy with VBL in KB-GRC1 cells (Shalinsky *et al.*, 1990*a*). Furthermore, both DPM and VPL alone produced a superior level of synergy with VBL compared to that for VP-16 or colchicine (Shalinsky *et al.*, 1990*a*), suggesting that these modulators may have a better potential for an improved chemotherapeutic response with VBL. We conclude that while DPM does not absolutely require the presence of a functioning P-glycoprotein to produce synergy with VBL in resistant cell lines, the presence of a functioning P-glycoprotein results in a much higher degree of synergy. If modulators of drug sensitivity are going to have a significant clinical impact, than high levels of synergy will likely be required. It therefore appears that DPM is unlikely to be a useful modulator for the treatment of multidrug resistant neoplasms that lack P-glycoprotein expression, but has more promise for the treatment of *mdr*1-expressing multidrug resistant tumours.

We thank Dr Igor B. Roninson for generously supplying the KB cell lines, Dr Marian B. Meyers for the immunoprecipitation labelling of the 2008 cells with the HYB-241 antibody and Dr Michael Andreeff for performing the flow cytometry of the 2008 cells with the HYB-241 antibody. Appreciation is also expressed to Mr Dennis Heath for expert HPLC analysis of VBL and to Boehringer Ingelheim Pharmaceuticals, Inc. for the generous gift of DPM.

D.R.S. was supported in part by grant CA 09290 from the National Institutes of Health. S.B.H. was supported by grants CA 23100 from the National Institutes of Health, grant CH 369 from the American Cancer Society, and grants from Boehringer Ingelheim Inc. and Bristol-Meyers, Co. This work was conducted in part by the Clayton Foundation for Research - California Division. Dr. Howell is a Clayton Foundation Investigator.

### References

ASOH, K.-I., SABURI, Y., SATO, S.-I., NOGAE, I., KOHNO, K. & KUWANO, M. (1989). Potentiation of some anticancer agents by dipyridamole against drug-sensitive and drug-resistant cell lines. *Jpn. J. Cancer Res.*, **80**, 475.

BAAS, F., JONGSMA, A.P.M., BROXTERMAN, H.J. & 7 others (1990). Non-P-glycoprotein-mediated mechanism for multidrug resistance precedes P-glycoprotein expression during in vitro selection for doxorubicin resistance in a human cancer cell line. *Cancer Res.*, **50**, 5392.

BECK, W.T., CIRTAIN, M.C. & LEFKO, J.L. (1983). Energy-dependent reduced drug binding as a mechanism of alkaloid resistance in human leukemic lymphoblasts. *Mol. Pharmacol.*, **24**, 485.

CHOI, K., CHEN, C., KREIGLER, M. & RONINSON, I. (1988). An altered pattern of cross-resistance in multi-drug resistant human cells results from spontaneous mutation in the mdr1 (P-glycoprotein) gene. *Cell*, **53**, 519.

CHOU, T.-C. & TALALAY, P. (1984). Quantitative analysis of dose-effect relationships: the combined effects of multiple drugs or enzyme inhibitors. *Adv. Enz. Reg.*, **22**, 27.

CHOU, J. & CHOU, T.-C. (1986). In *Dose-Effect Analysis with Microcomputers*. Pub. by Elsevier Science Publishers BV, Amsterdam, The Netherlands; distrib. by Elsevier-BIOSOFT, Cambridge, UK.

COLEY, H.M., WORKMAN, P. & TWENTYMAN, P.R. (1991). Retention of activity by selected anthracyclines in a multidrug resistant human large cell lung carcinoma line without P-glycoprotein hyperexpression. *Br. J. Cancer*, **63**, 351.

CORNWELL, M.M., PASTAN, I. & GOTTESMAN, M.M. (1987). Certain calcium channel blockers bind specifically to multidrug-resistant human KB carcinoma membrane vesicles and inhibit drug binding to P-glycoprotein. *J. Cell. Biol.*, **262**, 2166.

DANKS, M.K., YALOWICH, J.C. & BECK, W.T. (1987). Atypical multidrug resistant in a human leukemic cell line selected for resistance to teniposide (VM-26). *Cancer Res.*, **47**, 1297.

ENDICOTT, J.A. & LING, V. (1989). The biochemistry of P-glycoprotein-mediated multidrug resistance. *Ann. Rev. Biochem.*, **58**, 137.

FITZGERALD, G.A. (1987). Drug therapy with dipyridamole. *N. Engl. J. Med.*, **316**, 1247.

HARKER, W.G. & KADATZ, R.A. (1983). Mechanism of action of dipyridamole. *N. Engl. J. Med.*, **316**, 1247.

HARKER, W.G., SLADE, D.L., DALTON, W.S., MELTZER, P.S. & TRENT, J.M. (1989). Multidrug resistance in mitoxantrone-selected HL60 leukemia cells in the absence of P-glycoprotein overexpression. *Cancer Res.*, **49**, 4542.

HARKER, W.G., BAUER, D., ETIZ, B.B., NEWMAN, R.A. & SIKIC, B.I. (1986). Verapamil-mediated sensitization of doxorubicin-selected pleitropic resistance in human sarcoma cells: selectivity for drugs which produce human sarcoma cells: selectivity for drugs which produce DNA scission. *Cancer Res.*, **46**, 2369.

HOWELL, S.B., VICK, J., ANDREWS, P.A., VELURY, S. & SANGA, R. (1987). Biochemical modulation of cisplatin. In *Platinum and Other Metal Coordination Compounds in Cancer Chemotherapy*, M. Nicolini (ed.). pp. 228–234.

HOWELL, S.B., HOM, D.K., SANGA, R., VICK, J.S. & CHAN, T.C.K. (1989*a*). Dipyridamole enhancement of etoposide cytotoxicity. *Cancer Res.*, **49**, 4147.

HOWELL, S.B., HOM, D.K., SANGA, R., VICK, J.S. & ABRAMSON, I.A. (1989*b*). Comparison of the synergistic potentiation of etoposide, doxorubicin and vinblastine cytotoxicity by dipyridamole. *Cancer Res.*, **49**, 3178.

JURANKA, P.F., ZASTAAWNY, R.L. & LING, V. (1989). P-glycoprotein: multidrug resistance and a superfamily of membrane-associated transport proteins. *Faseb J.*, **?**, 2583.

KUSUMOTO, H., MAEHARA, Y., ANAI, H., KUSUMOTO, T. & SUGIMACHI, K. (1988). Potentiation of adriamycin cytotoxicity by dipyridamole against HeLa cell *in vitro* and sarcoma 180 cells *in vivo*. *Cancer Res.*, **48**, 1208.

LULIANO, L., VIOLI, F., GHISELLI, A., ALESANDRI, C. & BALSANO, F. (1989). Dipyridamole inhibits lipid peroxidation and scavenges oxygen radicals. *Lipids*, **24**, 430.

MARSH, W. & CENTER, M.S. (1987). Adriamycin resistance in HL60 cells and accompanying modification of a membrane protein contained in drug-sensitive cells. *Cancer Res.*, **47**, 5080.

MEYERS, M.G., RITTMAN-GRAUER, L., O'BRIEN, J.P. & SAFA, A.R. (1989). Characterization of monoclonal antibodies recognizing a $M_r$ 180,000 P-glycoprotein: differential expression of the $M_r$ 180,000 and $M_r$ 170,000 P-glycoproteins in multidrug-resistant human tumor cells. *Cancer Res.*, **49**, 3209.

MIRSKI, S.E.L., GERLACH, J.H. & COLE, S.P.C. (1987). Multidrug resistance in a small cell lung carcinoma cell line selected in Adriamycin. *Cancer Res.*, **47**, 2594.

PLAGEMANN, P.G.W., WOHLUETER, R.M. & WOFFENDIN, C. (1988). Nucleoside and nucleobase transport in animal cells. *Biochem. Biophys. Acta.*, **947**, 405.

RAMU, N. & RAMU, A. (1989). Circumvention of adriamycin resistance by dipyridamole analogues: a structure activity relationship study. *Int. J. Cancer*, **43**, 487.

RASHEED, S., NELSON-REES, W.A., TOTH, E.M., ARNSTEIN, P. & GARDNER, M.B. (1974). Characterization of a newly derived human sarcoma cell line (HT1080). *Cancer*, **33**, 1027.

RONINSON, I. (1991). Molecular genetic analysis of P-glycoprotein function. *American Association for Cancer Research Special Conference on Membrane Transport in Multidrug Resistance, Development and Disease*, Banff, Alberta, Canada, March 10–13.

SAFA, A. (1988). Photoaffinity labeling of the multidrug-resistance-related P-glycoprotein with photoactive analogs of verapamil. *Proc. Natl Acad. Sci.*, **85**, 7187.

SHALINSKY, D.R., ANDREEFF, M. & HOWELL, S.B. (1990*a*). Modulation of drug sensitivity by dipyridamole in multidrug resistant tumor cells *in vitro*. *Cancer Res.*, **50**, 7537.

SHALINSKY, D.R., CHRISTEN, R.D. & HOWELL, S.B. (1990*b*). The effect of dipyridamole and verapamil on the cellular pharmacology of vinblastine in multidrug resistant and sensitive tumor cells. *Proc. Am. Assoc. Cancer Res.*, **31**, 260.

SIROTNAK, F.M., YANG, C.-H., MINES, L.S., ORIBE, E. & BIEDLER, J.L. (1986). Markedly altered membrane transport and intracellular binding of vincristine in multidrug-resistant Chinese hamster cells selected for resistance to Vinca Alkaloids. *J. Cell. Phys.*, **126**, 266.

SLATER, L.M., MURRAY, S.L., WETZEL, M.W., SWEET, P. & STUPECKY, M. (1986). Verapamil potentiation of VP-16-213 in acute lymphatic leukemia and reversal of pleiotropic drug resistance. *Cancer Chemother. Pharmacol.*, **16**, 50.

SLOVAK, M.L., HOELTGE, G.A., DALTON, W.S. & TRENT, J.M. (1988). Pharmacological and biological evidence for differing mechanisms of doxorubicin resistance in two human tumor cell lines. *Cancer Res.*, **48**, 2793.

SLOVAK, M.L., MIRSKI, S.E.L., COLE, S.P.C., GERLACH, G.H. & YOHEM, K.H. & TRENT, J.M. (1991). Tumorigenic multidrug-resistant HT1080 cells do not overexpress receptors for epidermal growth factor. *Br. J. Cancer*, (in press).

THIMMAIAH, K.N. & SETHI, V.S. (1985). Chemical characterization of the degradation products of vinblastine dihydrogen sulfate. *Cancer Res.*, **45**, 5382.

TSURUO, T., LIDA, H., TSUKAGOSHI, S. & SAKURAI, Y. (1981). Overcoming of vinblastine resistance in P388 leukemia *in vivo* and *in vitro* through enhanced cytotoxicity of vincristine and vinblastine by verapamil. *Cancer Res.*, **41**, 1967.